# Treatment of Hemorrhoids by Chrysarobine

**Published:** 1890-03

**Authors:** 


### Selections.

### TREATMENT OF HEMORRHOIDS BY CHRYSAROBINE.

When the hemorrhoids are external:

| | |
|---|---|
| ℞—Chrysarobine | 80 centigr. |
| Iodoform | 30 " |
| Ext. Belladonnæ | 60 " |
| Vaselini | 25 Grammes. |
| M. | |

Sig. Externally.—Apply a small quantity to the hemorrhoids several times daily, bathing the parts beforehand with a 1 per cent. solution of phenic acid.

Internal hemorrhoids:

| | |
|---|---|
| ℞—Chrysarobine | 8 centigr. |
| Iodiform | 2 " |
| Ext. Belladonnæ | 1 " |
| Cacao Butter | 2 grammes. |
| M. | |

Fiat Suppository.—After three or four days the pains and hemorrhages will disappear, and after two or three months a recovery is made. Kossobudski, in *Semaine Med.*

